# Quantification of blood-based neurovascular unit biomarkers: an analytical platform comparison study

**DOI:** 10.1016/j.resplu.2026.101487

**Published:** 2026-09-11

**Authors:** Chloe P. Allen, Dorothée J. Goulet, Sophie Stukas, Andrew M. Agbay, Tessa F. Morelli, Tali Romero, Veronica Hirsch‐Reinshagen, Cheryl L. Wellington, Mypinder S. Sekhon, Jordan D. Bird

**Affiliations:** a Division of Critical Care Medicine, Department of Medicine, Vancouver General Hospital, West 12th Avenue, University of British Columbia, Vancouver, BC, Canada; b Department of Pathology and Laboratory Medicine, Vancouver General Hospital, West 12th Avenue, University of British Columbia, Vancouver, BC, Canada; c Djavad Mowafaghian Centre for Brain Health, Department of Pathology and Laboratory Medicine, Faculty of Medicine, University of British Columbia, Vancouver, BC, Canada; d School of Biomedical Engineering, University of British Columbia, 2332 Main Mall, Vancouver, British Columbia V6T 1Z3, Canada; e International Collaboration on Repair Discoveries, University of British Columbia, 818 W 10th Ave, Vancouver, British Columbia V5Z 1M9, Canada

**Keywords:** GFAP, Post-cardiac arrest brain injury, Hypoxic ischemic brain injury, Blood-based neurologic biomarkers, Neurofilament light-chain, UCH-L1

## Abstract

**Background:**

Hypoxic ischemic brain injury (HIBI) remains a significant cause of morbidity and mortality in patients following cardiac arrest. Many international guidelines for HIBI prognostication advocate for a multimodal approach with inclusion of blood-based biomarkers. However, advancements in analytical technology have increased uncertainty in selecting the optimal quantification tool and impeded application of these results across platforms in accordance with amalgamated prognostication thresholds.

**Methods:**

We conducted a *post hoc* analysis of a prospective observational cohort study in 32 critically ill patients undergoing withdrawal of life-sustaining therapies. Serum and plasma glial fibrillary acidic protein (GFAP), neurofilament-light (Nf-L), tau, and ubiquitin carboxyl hydrolase L-1 (UCH-L1) were quantified on the *Simoa HD-X* and *ARGO HT* analytical platforms respectively. Plasma GFAP and UCH-L1 samples were additionally quantified using the point-of-care *i-STAT Alinity.*

**Results:**

GFAP, Nf-L, Tau, and UCH-L1 were highly correlated between the *ARGO HT* and *Simoa HD-X* analytical platforms (all Pearson’s *r* ≥ 0.97, *P* ≤ 4.5e−13). In contrast, absolute agreement between the *i-STAT Alinity* and *Simoa HD-X* platforms was poor-to-moderate for GFAP (Intraclass correlation coefficient; ICC: 0.25 [−0.14, 0.59], *P* = 0.11) and poor-to-good for UCH-L1 (ICC: 0.60 [0.20, 0.82], *P* = 0.0026). The *ARGO HT* demonstrated strong inter-analyzer correlation with the *i-STAT Alinity* for GFAP (*r* = 0.98, *P* = 6.2e−18) and moderate correlation for UCH-L1 (*r* = 0.66, *P* = 0.00022).

**Conclusion:**

Collectively, this cross-platform validation highlights the importance of device-specific analytical methods and prognostication thresholds for each analytical platform considered for use in clinical practice.

## Highlights

• NfL concentrations demonstrated excellent agreement between plasma and serum matrices.
• GFAP and UCH-L1 showed poor agreement between the *Simoa HD-X* and *i-STAT Alinity* platform.
• Platform-specific correction factors or diagnostic thresholds are needed.

## Introduction

Hypoxic ischemic brain injury (HIBI) is the primary cause of morbidity and mortality in patients following resuscitation from cardiac arrest.^1^ Survivors of HIBI experience diverse clinical outcomes ranging from full neurologic recovery to survival with severe disability, underscoring the importance of accurate prognostication. Considerable effort has been directed towards refining prognostication methods for HIBI patients.^2^, ^3^, ^4^ Guidelines advocate for a multimodal approach including consideration of blood-based biomarkers in selected patients depending on the jurisdiction.^3^, ^4^, ^5^ However, while amalgamated biomarker-specific prognostication thresholds exist,^4^, ^6^ there remains significant heterogeneity between studies for biomarker concentration thresholds and relation to outcomes.^6^ These findings likely reflect heterogeneity in outcome assessment (*e.g.,* neurological outcomes versus survival), differences in the use of systematic neuroprognostication approaches and variation in timing of biomarker sampling, collection methods, and analytical platforms which have evolved substantially over time.^3^, ^6^, ^7^, ^8^

Advances in analytical technologies have facilitated more efficient biomarker quantification than standard enzyme-linked immunosorbent assays (ELISA) by allowing the simultaneous analysis of multiple analytes while providing enhanced analytical sensitivity, improved detection and quantification limits, and reduced sample volume requirements.^9^, ^10^ In particular, single molecule array (SIMOA) technology has advanced HIBI prognostication research by enabling the detection of biomarkers at femtomolar concentrations, providing up to 1000-fold greater analytical sensitivity than conventional ELISA-based assays, depending on the analyte. While invaluable in advancing biomarker science, their use remains largely research-based, with clinical assays and point-of-care platforms beginning to enter clinical practice. A key knowledge gap in the field relates to whether biomarker concentrations and thresholds agree across platforms.

The increasing number of bioassays and analytical platforms used to quantify the same biomarkers has heightened uncertainty regarding optimal assay selection and underscored the challenges of harmonizing results and prognostic thresholds across technologies.^9^ Establishing agreement across analytical platforms is essential to enable the development of platform-specific prognostic thresholds. Accordingly, rigorous validation of assays in clinical samples is urgently needed, particularly given the high-stakes nature of decisions regarding withdrawal of life-sustaining therapies (WLST), which may be informed by biomarker concentration thresholds.

In this study, we sought to investigate the agreement of blood-based neurovascular unit biomarker measurements across analytical platforms. Specifically, our objectives were: (1) to evaluate the agreement in biomarker concentrations between the Abbott i-STAT Alinity and the Quanterix Simoa HD-X platforms; and (2) to assess the correlation and agreement of biomarker concentrations between the Simoa HD-X and Alamar ARGO HT platforms, as well as between the Abbott i-STAT Alinity and Alamar ARGO HT platforms. We hypothesized that biomarkers used for HIBI prognostication would exhibit strong inter-platform agreement.

## Methods

### Study design and ethics

We conducted a *post hoc* analysis of a prospective observational cohort study in critically ill patients undergoing WLST.^11^ This study was conducted in accordance with reporting recommendations for agreement studies.^12^ The study was registered as a clinical study (NCT06130033). Ethics approval was obtained from the University of British Columbia Clinical Ethics Board (H23-00172).

### Bio-specimen sampling

Plasma and serum samples (Becton Dickinson® Vacutainer, cat. no. 367986) were obtained from an indwelling radial arterial catheter in patients with poor neurological prognosis. Sampling was obtained immediately prior to WLST. Following blood collection, vacutainers were inverted 5–10 times and placed in a dark room for 10 min in an enclosed container prior to being centrifuged at 600*g* for 10 min. The resulting serum and plasma were aliquoted into cryovials and immediately transferred to a −80°C freezer within 1 h of collection.

### Neurologic blood-based biomarkers measurements

Serum glial fibrillary acidic protein (GFAP), neurofilament-light (Nf-L), total tau, and ubiquitin carboxyl hydrolase L-1 (UCH-L1) were quantified using the Neurology-4-Plex-B Advantage Kit (cat ID 103345; lot 504005) on the *Simoa HD-X* platform. Each analyzer run contained an eight-point calibrator curve, two provided kit controls, three matrix controls, and up to 100 experimental samples all in duplicate; the average concentration of the values was used for analysis. Calibrator curve concentrations had an average absolute error of 3.2–6.4% and an average recovery of 100–101%. The average intra-plate coefficient of variation calculated using the patient samples for GFAP, NfL, total tau, and UCH-L1 serum samples were 5.5%, 5.0%, 4.5%, and 11.5%, respectively.

Plasma GFAP, Nf-L, brain-derived tau, and UCH-L1 were also quantified using the Neurology-4-Plex-D PLUS Advantage Kit (cat# 104822; lot 50300) on the *Simoa HD-X* platform. Calibrator curve concentrations had an average absolute error of 0.73–3.9%. The average intra-plate coefficient of variation calculated using the patient samples for GFAP, Nf-L, BD-tau, and UCH-L1 plasma samples were 3.8%, 3.4%, 3.1%, and 16%, respectively. Samples exceeding the upper limit of detection at the recommended 4-fold dilution were re-run at a 20-fold dilution and, if necessary, at a 100-fold dilution to bring concentrations within the assay analytical range. Given the known dilution linearity challenges for both tau and UCH-L1,^13^, ^14^, ^15^ all samples from a given patient were reanalyzed at the higher dilution factor to improve within-patient comparability. The *Simoa* assays were considered reference standard for this analysis, as it represents the most widely used commercially available platform in the literature evaluating outcome prediction after out-of-hospital cardiac arrest.^6^, ^16^, ^17^, ^18^, ^19^

Proteomic analysis of plasma samples to attomolar-level sensitivity was conducted using the NULISAseq™ CNS Disease Panel 120 (cat# 800104; lot 2425306) on the *ARGO HT* platform (Alamar Biosciences, CA, USA). Plasma samples were thawed from −80℃ and centrifuged at 10,000*g* for 10 min then subsequently, 25 µL of supernatant was loaded onto the assay plate. Assays were performed following the manufacturer’s instructions. The reporter DNA levels were quantified by Next-Generation Sequencing on the NextSeq2000 Sequencing System using a P2 XLEAP SBS Reagent Kit (100 cycles; Illumina Canada; cat no. 20101832). To normalize data, internal control (IC)-based normalization was applied by dividing the target counts for each sample well by the IC counts for that well. Inter-plate control (IPC) normalization was then performed by dividing the IC-normalized counts by the target-specific median values of the IPC (pooled plasma) sample replicates for that plate. Finally, the data were rescaled and log2-transformed to normalize distribution. Resulting values are referred to as NULISA Protein Quantification (NPQ) units. The plate-specific limit of detection for each target assay was determined by calculating the mean plus three standard deviations (SD) of the unlogged normalized counts obtained from negative control samples on each plate. Researchers performing the analysis were blinded to individual participant data. The coefficient of variation of internal control signals across control and critically ill patient samples were 11.7% and 12.2%, respectively. The coefficient of variation of the total assay signal across all inter-plate controls and critically ill patient samples were 11.7% and 1.0%, respectively.

Plasma samples were quantified for GFAP and UCH-L1 using the point-of-care *i-STAT* TBI Plasma Cartridges (04X64-25, Abbott Laboratories, IL, USA).^20^, ^21^ Calibration of GFAP and UCH-L1 throughout the reportable range was validated using the *i-STAT* Calibration Verification Set (cat ID 06P1724, lot 240124), frozen serum with a 3-level concentration range for each of GFAP and UCH-L1, generated by Abbott Laboratories. All test procedures were performed in accordance with the manufacturer’s instructions. Plasma specimens were analyzed in a randomized order and the investigator performing the analyses was blinded to participant status.

### Statistical analysis

All analyses were performed using R Statistical Software (v4.3.1; R Core Team) with *irr*^22^ and *rstatix*^23^ packages. Plasma samples with analytes that were above the upper limit of quantification or below the lower limit of quantification for one or more of the analytical platforms, but for which a numerical value was obtained, were included in the analysis (Fig. S1). Plasma samples with analytes that were above the upper limit of detection despite increased dilution were excluded. Relative agreement (*i.e.,* consistency) was defined as whether analyzers ordered subjects in the same rank order, even if the absolute quantification differed between analytical platforms. Owing to the ARGO HT NULISAseq CNS Disease Panel 120 reporting relative rather than absolute biomarker concentrations, the relationship between measurements was assessed using Pearson correlation coefficients rather than agreement analyses.^24^ Absolute agreement was defined as the extent to which two analytical platforms yielded interchangeable biomarker concentrations for the same blood sample. Absolute inter-platform agreement between the *i-STAT Alinity* and *Simoa HD-X* platforms was evaluated using two-way random effects single rater absolute agreement intraclass correlation coefficients (ICC).^25^ ICC values less than 0.5 indicate poor reliability, values between 0.5 and 0.75 indicate moderate reliability, values between 0.75 and 0.9 indicate good reliability, and values greater than 0.90 indicate excellent reliability.^25^ Absolute agreement was also assessed by mean biases and 95% limits of agreements.^26^, ^27^ Statistical significance was set at *P* < 0.05.

## Results

Thirty-two patients were included in this analysis with 26 of these study patients (81%) having measurements available for all three analytical platforms. Patient demographics are presented in Table 1.

**Table 1:** Patient demographics for patients undergoing withdrawal of life-sustaining measures.

**Demographics**	**Patients (*N* = 32)**
Age – years (mean ± SD)	60 ± 15
Female sex – no. (%)	7 (22%)
**Admission diagnosis – no. (%)**	
Hypoxic ischemic brain injury	14 (44%)
Sepsis	5 (16%)
Traumatic brain injury	5 (16%)
Intracerebral hemorrhage	5 (16%)
Other	3 (9%)
**Ethnicity – no. (%)**	
Caucasian	26 (81%)
African Canadian	2 (6%)
Indigenous	1 (3%)
South Asian	2 (6%)
Asian	1 (3%)
**Comorbidities – no. (%)**	
Opioid addiction	8 (25%)
Hypertension	10 (31%)

Data are presented as no. (%).

### Overall detectability

A total of *N* = 32 arterial plasma samples taken prior to WLST were available for analysis. All 32 blood samples were analyzed on the *Simoa HD-X* with 26 samples (81%) analyzed on the *ARGO HT,* and 27 (84%) analyzed on the *i-STAT Alinity.* Mean detectability across biomarkers (*i.e.,* values within limits of detection) for the analyzer was 97% for the *ARGO HT*, 74% for the *i-STAT Alinity*, and 78% for the *Simoa HD-X* (Fig. S1).

### Analytical comparison of serum and plasma on *Simoa HD-X*

GFAP had good-to-excellent agreement between serum and plasma (Fig. 1A) with serum concentrations being consistently greater than plasma concentrations by 1-fold (Fig. 1B). Nf-L had good-to-excellent agreement between serum and plasma (Fig. 1C) with concentrations not being matrix specific (Fig. 1D). UCH-L1 had poor-to-good agreement between serum and plasma (Fig. 1E) with absolute concentration being greater in serum than plasma (Fig. 1F).

**Fig. 1:**
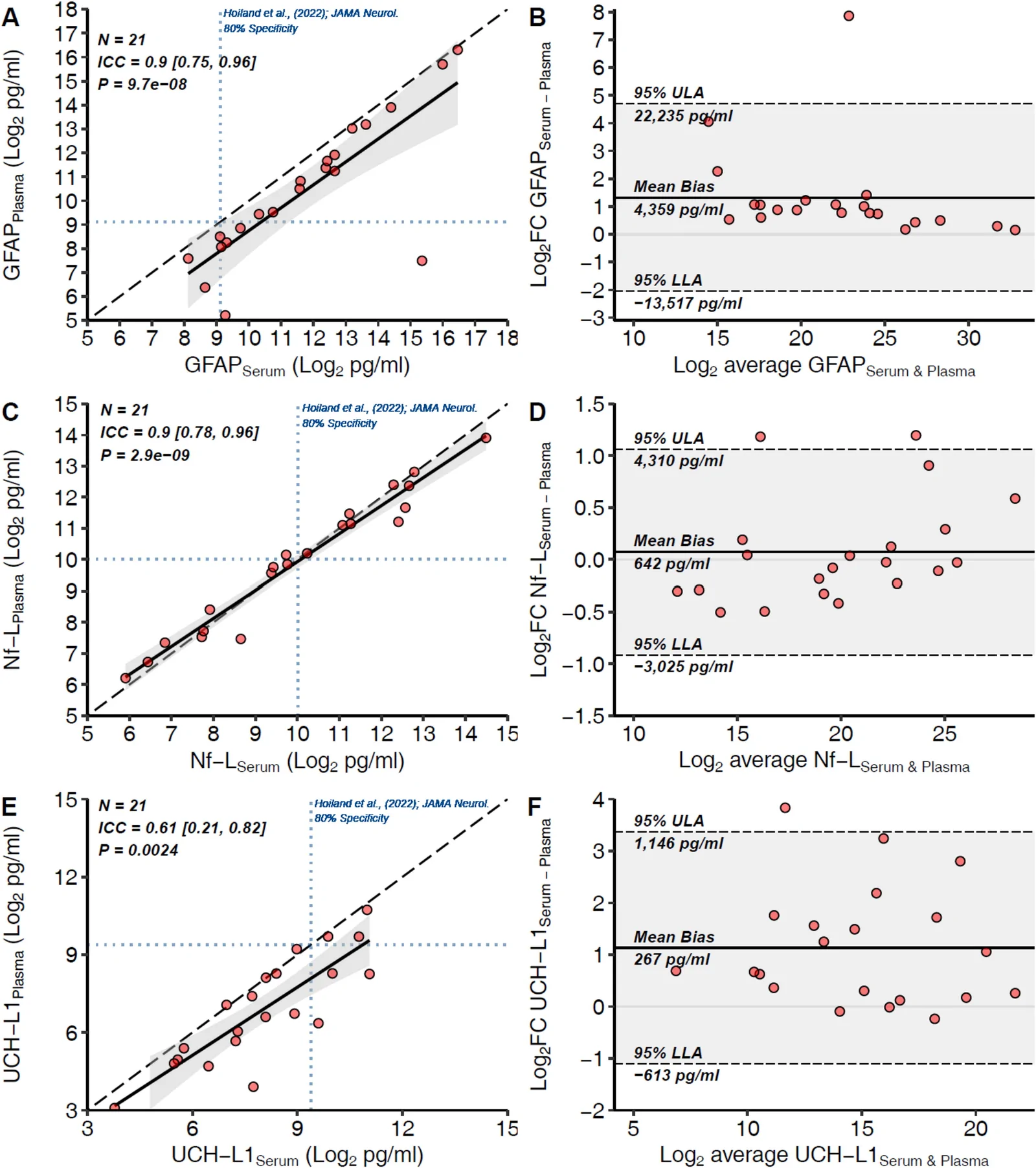
**Cross matrix comparison on the *Simoa HD-X***. Arterial plasma samples were analyzed using the Neurology-4-Plex-D PLUS Advantage Kit and arterial serum samples were analyzed using the Neurology-4-Plex-B Advantage Kit. Relative agreement was linearly group fit (solid black line) with 95% confidence intervals (gray ribbons) for GFAP (*A*), UCH-L1 (*C*). and Nf-L (*E*). The optimal threshold at 80% specificity for prognostication is annotated from Hoiland et al. (2022) (blue dotdash line). Bland–Altman plots for the comparisons between the average serum N4PB and plasma N4PD+ measurements for GFAP (*B*), UCH-L1 (*D*), and Nf-L (*F*) are shown along with the limits of agreement (dashed gray lines) and mean bias (black line) between assays. *Abbreviations:* GFAP, glial fibrillary acidic protein; Nf-L, neurofilament light chain; UCH-L1, ubiquitin C-terminal hydrolase L1; ICC, intraclass correlation coefficient; ULA, 95% upper limit of agreement; LLA, 95% lower limit of agreement. (For interpretation of the references to color in this figure legend, the reader is referred to the web version of this article.)

### Analytical comparison of *Simoa HD-X* and *i-STAT Alinity* platforms

GFAP (Fig. 2A) and UCH-L1 (Fig. 2C) demonstrated poor absolute agreement between the *Simoa HD-X* and *i-STAT Alinity* platforms in both the full cohort (Fig. 2) and the HIBI-specific cohort (Fig. S2). While GFAP measurements were within the reportable range of the *i-STAT Alinity* (74%), UCH-L1 measurements consistently fell within the lower range of detection with most biospecimens being below the reportable range (59%). Mean bias and 95% confidence intervals suggest the *i-STAT Alinity* underestimates absolute concentrations compared to the *Simoa HD-X* platform for both GFAP (Fig. 2B) and UCH-L1 (Fig. 2D).

**Fig. 2:**
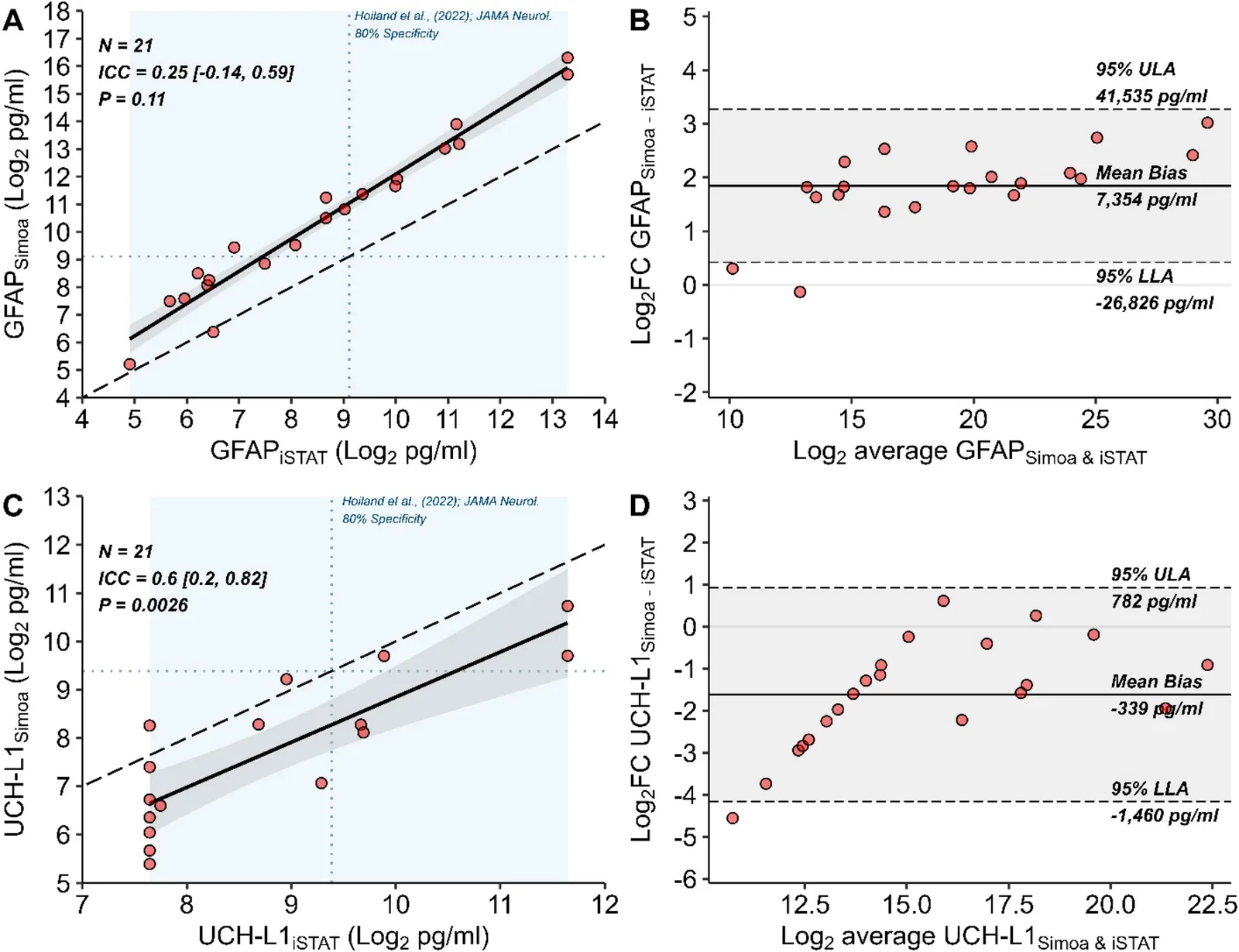
**Inter-analyzer absolute agreement between the *i-STAT Alinity* and *Simoa HD-X* analytical platforms**. Arterial plasma samples were analyzed using the Neurology-4-Plex-D PLUS Advantage Kit on the *Simoa HD-X* platform and using the point-of-care *i-STAT* TBI Plasma Cartridges on the *i-STAT Alinity* platform. Both the *i-STAT Alinity* and *Simoa HD-X* data are presented on a log2 scale for visualization. Relative agreement was linearly group fit (solid black line) with 95% confidence intervals (gray ribbons) for GFAP (*A*) and UCH-L1 (*C*) using intraclass correlation coefficients. The upper and lower ranges for detection of GFAP on the *i-STAT Alinity* are 30 pg/ml and 10,000 pg/ml (shaded light blue area; *A*). The upper and lower ranges for detection of UCH-L1 on the *i-STAT Alinity* are 200 pg/ml and 3200 pg/ml (shaded light blue; *C*). The optimal threshold at 80% specificity for prognostication is annotated from Hoiland et al. (2022) (blue dotdash line). Bland–Altman plots for the comparisons between the average *i-STAT Alinity* and *Simoa HD-X* measurements for GFAP (B) and UCH-L1 (D) are shown along with the 95% limits of agreement (dashed gray lines) and mean bias (black line) between analytical platforms. *Abbreviations:* GFAP, glial fibrillary acidic protein; Nf-L, neurofilament light chain; UCH-L1, ubiquitin C-terminal hydrolase L1; ICC, intraclass correlation coefficient; ULA, 95% upper limit of agreement; LLA, 95% lower limit of agreement. (For interpretation of the references to color in this figure legend, the reader is referred to the web version of this article.)

Using a GFAP prognostication threshold of 552 pg/mL in patients with HIBI,^6^ 10 of 12 patients (83%) would have exceeded the threshold when measured on the *Simoa HD-X* platform, compared with only 7 of 12 patients (58%) when measured on the point-of-care *i-STAT Alinity* platform (Fig. S2).

### Analytical comparison of *ARGO HT* and *Simoa HD-X* platforms

GFAP, Nf-L, UCH-L1 and tau demonstrated very strong inter-analyzer correlation between the gold-standard *ARGO HT* and *Simoa HD-X* analytical platforms (Fig. 3 and Fig. S3).

**Fig. 3:**
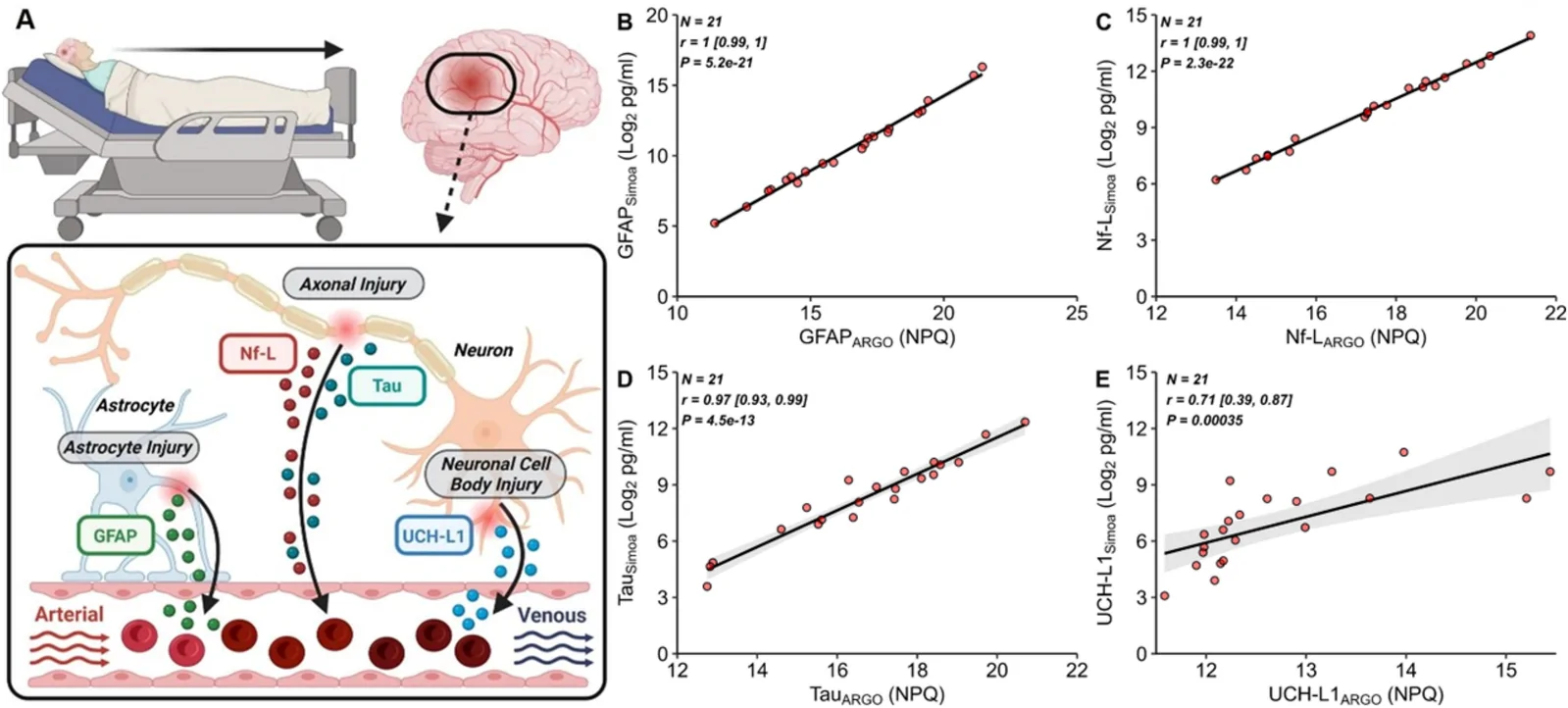
**Inter-analyzer correlation between the *Simoa HD-X* and the *ARGO HT* analytical platforms**. Arterial plasma samples were analyzed using the NULISAseq™ CNS Disease Panel 120 panel on the *ARGO HT* platform and using the Neurology-4-Plex-D PLUS Advantage Kit on the *Simoa HD-X* platform. Neurovascular unit biomarkers (*A*) consisted of GFAP (*B*), Nf-L (*C*), total Tau (*D*), and UCH-L1 (*E*). *Simoa* data was log2 transformed to allow for comparison to *ARGO HT* NULISA data. Relative agreement was linearly group fit (solid black line) with 95% confidence intervals (gray ribbons) using Pearson R correlations. *Abbreviations:* GFAP, glial fibrillary acidic protein; Nf-L, neurofilament light chain; UCH-L1, ubiquitin C-terminal hydrolase L1; NPQ, NULISA protein quantification; UCH-L1, ubiquitin C-terminal hydrolase L1. Panel A was created in BioRender. Bird, J. (2026) https://BioRender.com/3kyamki.

### Analytical comparison of *ARGO HT* and *i-STAT Alinity* platforms

GFAP demonstrated very strong inter-analyzer correlation between the *ARGO HT* and the *i-STAT Alinity* analytical platforms, while UCH-L1 exhibited moderate inter-analyzer correlation (Fig. 4 and Fig. S4).

**Fig. 4:**
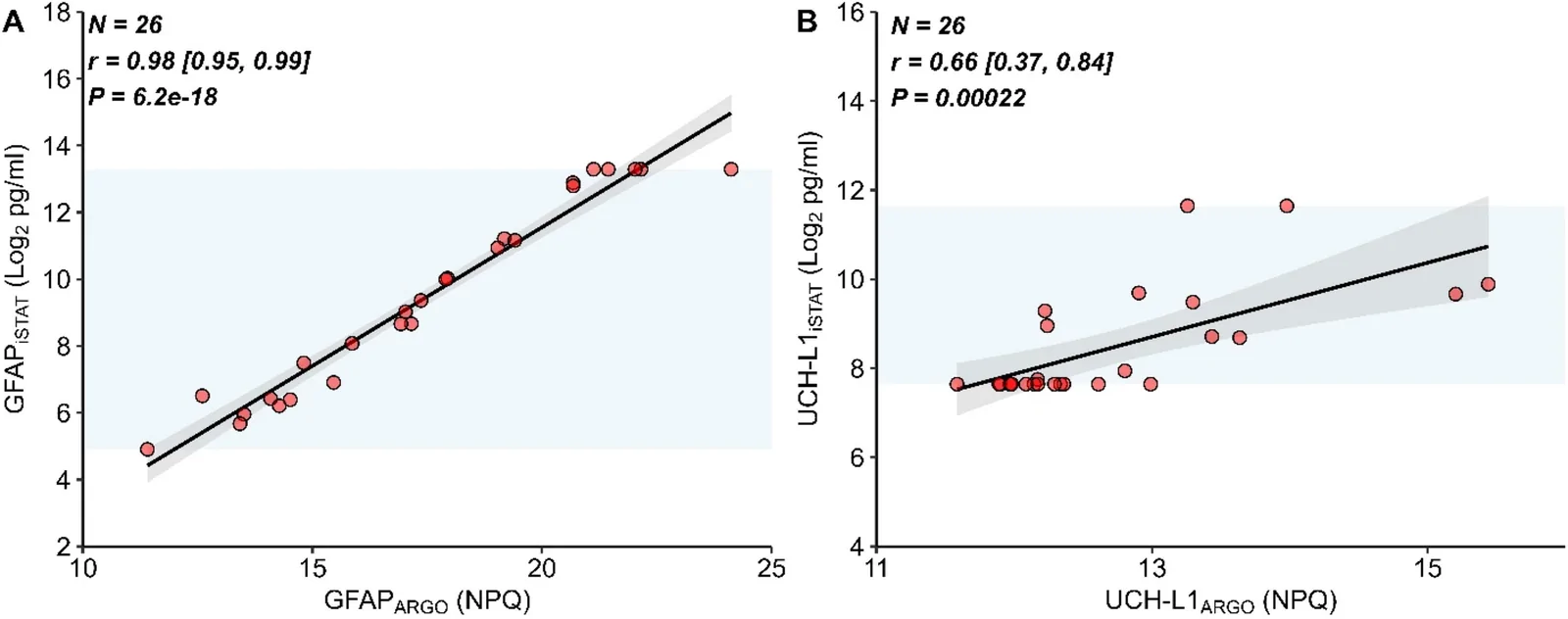
**Inter-analyzer correlation between the *i-STAT Alinity* and *ARGO HT* analytical platforms**. Arterial plasma samples were analyzed using both the NULISAseq™ CNS Disease Panel 120 panel on the *ARGO HT* platform and using the point-of-care i-STAT TBI Plasma Cartridges on the *i-STAT Alinity* platform. The *i-STAT Alinity* data was log2 transformed to allow for comparison to *ARGO HT* NULISA data. Relative agreement was linearly group fit (solid black line) with 95% confidence intervals (gray ribbons) using a Pearson R correlation. Upper and lower ranges for detection on the *i-STAT Alinity* (shaded blue area) for GFAP (*A*) and UCH-L1 (*B*) are depicted. *Abbreviations:* GFAP, glial fibrillary acidic protein; UCH-L1, ubiquitin C-terminal hydrolase L1. (For interpretation of the references to color in this figure legend, the reader is referred to the web version of this article.)

## Discussion

We conducted a *post hoc* analysis of a clinical study to evaluate the correlation and agreement of neurovascular unit blood-based biomarker measurements across three analytical platforms. Despite reasonable inter-platform correlations for GFAP, Nf-L, Tau, and UCH-L1, absolute agreement in biomarker concentrations was limited. These findings highlight the importance of evaluating agreement between analytical platforms as neurologic prognostication thresholds are likely to be analyzer specific. Methods to harmonize biomarker measurements across platforms will be important for future studies assessing the utility of blood-based biomarkers for neurological prognostication and decisions regarding WLST.

We found that Nf-L concentrations were comparable between serum and plasma, suggesting that matrix-specific thresholds may not be required for the use of Nf-L in neuroprognostication. This finding may support the application of uniform Nf-L thresholds across matrices for the prediction of poor neurological outcome with high specificity.^1^, ^18^, ^28^

Neurological prognostication following HIBI relies on a multimodal approach combining clinical examination, neuroimaging, and electrophysiological assessments, with blood-based biomarkers increasingly recommended as an adjunctive tool in selected patients.^3^, ^4^ Blood-based biomarkers offer the advantage of objective and potentially generalizable numerical thresholds that are not dependent on expert interpretation, unlike electrophysiology or neuroimaging, and are not confounded by sedative exposure, unlike clinical examination^7^. While invaluable, clinicians should recognize the limitations of blood-based biomarker prognostication. Foremost, the analytical sensitivity, quantification thresholds, and analytical methods used in laboratory medicine are often not equivalent across platforms, necessitating the development of platform-specific thresholds or correction factors.

The proliferation of available analytical platforms and bioassays has driven significant progress in the emerging field of neurological blood-based biomarkers. However, despite increased accessibility and reductions in cost, the reliability and reproducibility of these measurements have remained relatively uninformed. Studies that compare biomarkers measurements often fail to distinguish between consistency of measurement (*i.e.,* correlation) from agreement of measurement (*i.e.,* comparability of absolute concentrations between platforms). This distinction is of particular interest in HIBI, where the primary clinical application of blood-based biomarkers is the use of predefined prognosis thresholds.^28^ The validity and transferability of these thresholds depend on inter-analyzer agreement rather than inter-analyzer consistency.

While neural specific enolase (NSE) is currently the only blood based biomarker recommended in international guidelines for post cardiac arrest neuroprognostication,^2^, ^3^, ^28^ considerable uncertainty remains regarding optimal prognosis thresholds and substantial variability in reported prognostic accuracy persists.^29^ In 2006, NSE was recommended as the only biomarker by the American Academy of Neurology guidelines^30^ with a cut-off level of 33 ng/ml for prediction of outcome in comatose survivors after cardiopulmonary resuscitation. However, in 2015, the ERC/ESCIM guidelines recommended the use of locally established NSE “high levels” to predict poor neurological outcome, largely informed by the biomarker substudy of the TTM-trial.^31^ In 2021, the ERC/ESICM guidelines subsequently refined their recommendation by specifying a NSE cut-off level of 60 ng/ml for neuroprognostication, following studies that reported widely varying thresholds to achieve 100% specificity and a false positive rate up to 29.3% when using the previously proposed 33 ng/mL threshold.^4^, ^32^, ^33^, ^34^, ^35^, ^36^ The lack of a commercially available Simoa-based NSE assay coupled with evidence suggesting the superior prognostic performance of Nf-L for predicting poor outcome after cardiac arrest,^17^, ^19^, ^29^, ^37^, ^38^, ^39^ formed the basis for our decision not to include NSE in the present analysis. However, the history of NSE-guided neuroprognostication highlights the necessity of rigorous validation of biomarker analytical platforms before clinical implementation, as insufficient standardization and validation can result in shifting prognostic thresholds and changing interpretations of biomarker performance.

As Nf-L has progressed from a research biomarker to a candidate laboratory test for prognostic use, the Roche Elecsys® and Fujirebio Lumipulse® are among the most likely platforms to achieve future clinical implementation, with CE marking for *in-vitro* diagnostics.^40^ The Siemens Healthineers Atellica®IM, which is certified for routine diagnostics, also possesses the first Nf-L assay with Health Canada licensing.^41^ Notably, prior investigations of Nf-L immunoassay comparability between the *Simoa*, *Elecsys*, *Lumipulse*, and *Atellica* have demonstrated bias, amplified by differences in antibodies and detection methods.^42^, ^43^ Such differences may preclude the direct comparability of results between assays for clinical purposes and necessitate assay specific interpretation for routine use.^42^, ^44^ While our inability to include these platforms within our methodological comparison limits the robustness of our results, we provide a generalizable design framework that can be implemented in future studies, while highlighting the need for further standardization and harmonization as new analytical platforms and assays emerge.

From our study, the absolute biomarker concentrations measured by the *Simoa HD-X* and the *i-STAT Alinity* were not directly comparable, consistent with previous analyses of GFAP across these platforms.^44^ Accordingly, the integration of blood-based biomarkers into routine clinical practice should be preceded by validation and calibration of new analytical platforms against an established reference standard, ideally the platform used to derive existing prognosis thresholds.^18^ Such validation should include the comparison of identical blood samples across the analytical measurement range of each assay.^45^ If adequate agreement is demonstrated, platform-specific thresholds or harmonization factors can be developed, as has recently been described for plasma p-tau217 assays in Alzheimer’s disease.^46^ Combined with inter-laboratory reproducibility studies, these steps may facilitate the clinical translation of blood-based biomarkers for HIBI.^47^

Moving forward, resuscitation scientists can play an important role in supporting the validation of new biomarker assays and analytical platforms. Prospective biobanking initiatives provide well-characterized samples spanning a broad analytical range that can be leveraged for assay validation and allow for harmonization of studies. As additional platforms are validated, cross-platform comparison may facilitate consolidation of the existing literature and improve transferability of prognostic thresholds. Finally, early validation of emerging point-of-care devices such as the *i-STAT Alinity* may accelerate the adoption of biomarker-guided neuroprognostication in resource-limited and out-of-hospital settings.

The Target Temperature Management after out-of-hospital cardiac arrest (TTM) trials^48^, ^49^, ^50^ have been fundamental to advancing large-scale biomarker research and defining their utility for predicting neurological outcome.^48^ Beyond establishing that NSE concentrations are not significantly influenced by temperature management, the TTM trials and biomarker sub-studies have been instrumental in identifying and validating alternative biomarkers including Nf-L, tau, and GFAP for earlier and more accurate neurological prognostication. The prospective collection of biospecimens from patients at multiple centers at serial timepoints after return of spontaneous circulation provides a robust biobank, which provides a unique opportunity for *post hoc* inter-platform validation studies, supporting the establishment and standardization of biomarker thresholds used to inform WLST decisions.

This study has several limitations. First, the small sample size limited statistical power and precision around agreement estimates, particularly for subgroup analyses. Second, the heterogeneous cohort, which included patients with different forms of neurological injury, may limit generalizability to specific populations. Third, not all biomarkers were measurable across all platforms, limiting direct comparison between assays. Fourth, the study was conducted at a single center, and inter-laboratory reproducibility could not be assessed. Finally, although this study assessed analytical agreement and correlation, it was not designed to evaluate prognostic performance or derive platform-specific clinical thresholds.

## Conclusion

Our results suggest poor agreement between the *Simoa HD-X* and *i-STAT Alinity* platforms for both GFAP and UCH-L1 in a cohort of critically ill patients undergoing WLST. In contrast, the *Simoa HD-X* and *ARGO-HT* platforms demonstrated strong inter-platform correlation for Nf-L, GFAP, and tau measurements. These preliminary findings highlight important analytical differences between platforms that may influence threshold-based neuroprognostication and clinical decision-making. Platform-specific validation, thresholds, and potentially harmonization or correction factors will likely be required before biomarker measurements can be used interchangeably across analytical systems.

## Funding source

This study was funded by operating grants from the Vancouver General Hospital and UBC Hospital Foundation. C.P.A. was funded through a Canadian Institute of Health Research – Master’s Award (#6556). J.D.B. was funded through a Canadian Institute of Health Research – Doctoral Award (#187576).

## CRediT authorship contribution statement

**Chloe P. Allen:** Writing – review & editing, Writing – original draft, Visualization, Software, Methodology, Investigation, Formal analysis, Data curation. **Dorothée J. Goulet:** Writing – review & editing, Supervision, Project administration, Methodology, Investigation, Data curation. **Sophie Stukas:** Writing – review & editing, Supervision, Resources, Project administration, Methodology, Formal analysis, Data curation. **Andrew M. Agbay:** Writing – review & editing, Investigation, Formal analysis, Data curation. **Tessa F. Morelli:** Writing – review & editing, Resources, Formal analysis, Data curation. **Tali Romero:** Writing – review & editing, Investigation, Formal analysis, Data curation. **Veronica Hirsch‐Reinshagen:** Writing – review & editing, Supervision, Project administration, Investigation. **Cheryl L. Wellington:** Writing – review & editing, Supervision, Project administration, Methodology. **Mypinder S. Sekhon:** Writing – review & editing, Writing – original draft, Visualization, Validation, Supervision, Resources, Project administration, Methodology, Investigation, Funding acquisition, Formal analysis, Data curation, Conceptualization. **Jordan D. Bird:** Writing – review & editing, Visualization, Supervision, Software, Project administration, Investigation, Formal analysis, Data curation, Conceptualization.

## Declaration of competing interest

M.S.S. serves as a member of the Editorial Board for Resuscitation. In line with the journal’s conflict of interest policies, M.S.S. is recused from all aspects of the editorial and peer review process for this systematic review. The submission was handled independently by other editors with no involvement from M.S.S.

## Appendix A Supplementary material

Supplementary Data 1 The following are the Supplementary material to this article which provide a summary of analytical platforms and further assessment of inter-analyzer bias.
