# Supplementary material for: Quantification of blood-based neurovascular unit biomarkers: an analytical platform comparison study
Source: Resusc Plus. 2026 Sep 11;32:101487. doi: 10.1016/j.resplu.2026.101487 (PMC13629343; doi:10.1016/j.resplu.2026.101487)
Supplement: Supplementary file 1 [file mmc1.docx]

**Quantification of Blood-Based Neurovascular Unit Biomarkers:**

**An Analytical Platform Comparison Study**

**Supplementary Appendix**

***Authors***

Chloe P. Allen^1^, Dorothée J. Goulet^1^, Sophie Stukas^1,3^, Andrew M. Agbay^3^, Tessa F. Morelli^3^, Tali Romero^,3^, Veronica Hirsch‐Reinshagen^2,3,5^, Cheryl L. Wellington^3,4,5^, Mypinder S. Sekhon^*1,3,5^, Jordan D. Bird^*1^

***Affiliations***	^1^Division of Critical Care Medicine, Department of Medicine, Vancouver General Hospital, West 12th Avenue, University of British Columbia, Vancouver, BC, Canada

^2^Department of Pathology and Laboratory Medicine, Vancouver General Hospital, West 12^th^ Avenue, University of British Columbia, Vancouver, BC, Canada

^3^Djavad Mowafaghian Centre for Brain Health, Department of Pathology and Laboratory Medicine, Faculty of Medicine, University of British Columbia, Vancouver, BC, Canada

^4^School of Biomedical Engineering, University of British Columbia, 2332 Main Mall, Vancouver, British Columbia, Canada, V6T 1Z3.

^5^International Collaboration on Repair Discoveries, University of British Columbia, 818 W 10th Ave, Vancouver, British Columbia, Canada, V5Z 1M9.

***Corresponding Author(s)***

Dr. Mypinder S. Sekhon MD PhD	Jordan D. Bird MSc

Critical Care Medicine	Critical Care Medicine

Vancouver General Hospital	Vancouver General Hospital

Room 2438, Jim Pattison Pavilion, 2nd Floor	Room 2438, Jim Pattison Pavilion, 2^nd^ Floor

855 West 12th Avenue	855 West 12^th^ Avenue

Vancouver BC	V5Z 1M9	Vancouver BC	V5Z 1M9

Phone: 604-875-5949	Phone: 587-577-7159

Fax: 604-875-5957	Email: [Jordan.bird@alumni.ubc.ca](mailto:Jordan.bird@alumni.ubc.ca)

Email: [mypindersekhon@gmail.com](mailto:mypindersekhon@gmail.com)

***Word Count***	2785/3000

**Keywords:**	*GFAP, post-cardiac arrest brain injury, hypoxic ischemic brain injury, blood-based neurologic biomarkers, neurofilament light-chain, UCH-L1*

* Mypinder S. Sekhon & Jordan D. Bird contributed equally as last author

**Figures**

**Supplementary Figure 1. Summary of analytical platforms.** Data are presented as no. (%) where applicable. The number of samples for each platform indicates the number of samples analyzed for each of the analytes indicated respectively. Abbreviations: GFAP, glial fibrillary acidic protein; Nf-L, neurofilament light chain; UCH-L1, ubiquitin C-terminal hydrolase L1; MAPT, microtubule associated protein tau; BD-tau, brain-derived tau; WLST, withdrawal of life sustaining therapies; ULOQ, upper limit of quantification; ULOD, upper limit of detection; LLOQ, lower limit of quantification.

**Supplementary Figure 2. Inter-analyzer absolute agreement between the *i-STAT Alinity* and *Simoa HD-X* analytical platforms for HIBI.** Arterial plasma samples were analyzed using the Neurology-4-Plex-D PLUS Advantage Kit on the *Simoa HD-X* platform and the point-of-care *i-STAT* TBI Plasma Cartridges on the *i-STAT Alinity* platform. Both the *i-STAT Alinity* and *Simoa HD-X* data for a cohort of subset of HIBI pateints are presented on a log2 scale for visualization. Relative agreement was linearly group fit (solid black line) with 95% confidence intervals (grey ribbons) for GFAP (*A*) and UCH-L1 (*C*). The upper and lower ranges for detection of GFAP on the *i-STAT Alinity* are 30 pg/ml and 10,000 pg/ml (shaded light blue area; *A*). The upper and lower ranges for detection of UCH-L1 on the *i-STAT Alinity* are 200 pg/ml and 3,200 pg/ml (shaded light blue; *C*). The optimal threshold at 80% specificity for prognostication is annotated from Hoiland *et al.,* 2022 (blue dotdash line). Bland–Altman plots for the comparisons between the average *Abbott i-STAT Alinity* and *Simoa HD-X* measurements for GFAP (B) and UCH-L1 (D) are shown along with the limits of agreement (dashed grey lines) and mean bias (black line) between instruments. Abbreviations: GFAP, glial fibrillary acidic protein; UCH-L1, ubiquitin C-terminal hydrolase L1; ICC, intraclass correlation coefficient.

**Supplementary Figure 3. Inter-analyzer bias between the *ARGO HT* and *Simoa HD-X* analytical platforms.** Arterial plasma samples were analyzed using the NULISAseq™ CNS Disease Panel 120 panel on the *ARGO HT* platform and using the Neurology-4-Plex-D PLUS Advantage Kit on the *Simoa HD-X* platform. *Simoa* data was log2 transformed to allow for comparison to Alamar NULISA data. Passing-Bablok regression (black dotted line) was computed for GFAP (*A*), Nf-L (*B*), total Tau (*C*), and UCH-L1 (*D*). A regression line corresponding to perfect agreement between the two measurement methods is indicated (dashed grey line). Abbreviations: GFAP, glial fibrillary acidic protein; Nf-L, neurofilament light chain; UCH-L1, ubiquitin C-terminal hydrolase L1.

**Supplementary Figure 4. Inter-analyzer proportional bias between the *ARGO HT* and *i-STAT Alinity* analytical platforms.** Arterial plasma samples were analyzed using the NULISAseq™ CNS Disease Panel 120 panel on the *ARGO HT* platform and the point-of-care *i-STAT* TBI Plasma Cartridges on the *i-STAT Alinity* platform. Data from the *i-STAT* was log2 transformed to allow for comparison to Alamar NULISA data. Passing-Bablok regression (black dotted line) was computed for GFAP (*A*) and UCH-L1 (*B*). The upper and lower ranges for detection of GFAP on the *i-STAT Alinity* are 30 pg/ml and 10,000 pg/ml (shaded light blue area; *A*). The upper and lower ranges for detection of UCH-L1 on the *i-STAT Alinity* are 200 pg/ml and 3,200 pg/ml (shaded light blue; *B*). A regression line corresponding to perfect agreement between the two measurement methods is indicated (dashed grey line). Abbreviations: GFAP, glial fibrillary acidic protein; UCH-L1, ubiquitin C-terminal hydrolase L1.
